# Mental Health Presentations Across Health Care Settings During the First 9 Months of the COVID-19 Pandemic in England: Retrospective Observational Study

**DOI:** 10.2196/32347

**Published:** 2022-08-03

**Authors:** Gillian E Smith, Sally E Harcourt, Uy Hoang, Agnieszka Lemanska, Alex J Elliot, Roger A Morbey, Helen E Hughes, Iain Lake, Obaghe Edeghere, Isabel Oliver, Julian Sherlock, Richard Amlôt, Simon de Lusignan

**Affiliations:** 1 Real-time Syndromic Surveillance Team Field Service UK Health Security Agency Birmingham United Kingdom; 2 National Institute for Health and Care Research Health Protection Research Unit in Emergency Preparedness and Response King's College London London United Kingdom; 3 Nuffield Department of Primary Care Health Sciences University of Oxford Oxford United Kingdom; 4 School of Environmental Science University of East Anglia Norwich United Kingdom; 5 National Institute for Health and Care Research Health Protection Research Unit in Behavioural Science and Evaluation Population Health Sciences University of Bristol Bristol United Kingdom; 6 Science Group UK Health Security Agency London United Kingdom; 7 Behavioural Science and Insights Unit UK Health Security Agency London United Kingdom; 8 Faculty of Health and Medical Sciences University of Surrey Surrey United Kingdom; 9 Royal College of General Practitioners London United Kingdom

**Keywords:** pandemic, public health, syndromic surveillance, mental health, anxiety, sleep problems, COVID-19, health care, health surveillance, health care service

## Abstract

**Background:**

The COVID-19 pandemic has resulted in an unprecedented impact on the day-to-day lives of people, with several features potentially adversely affecting mental health. There is growing evidence of the size of the impact of COVID-19 on mental health, but much of this is from ongoing population surveys using validated mental health scores.

**Objective:**

This study investigated the impact of the pandemic and control measures on mental health conditions presenting to a spectrum of national health care services monitored using real-time syndromic surveillance in England.

**Methods:**

We conducted a retrospective observational descriptive study of mental health presentations (those calling the national medical helpline, National Health Service [NHS] 111; consulting general practitioners [GPs] in and out-of-hours; calling ambulance services; and attending emergency departments) from January 1, 2019, to September 30, 2020. Estimates for the impact of lockdown measures were provided using an interrupted time series analysis.

**Results:**

Mental health presentations showed a marked decrease during the early stages of the pandemic. Postlockdown, attendances for mental health conditions reached higher than prepandemic levels across most systems—a rise of 10% compared to that expected for NHS 111 and 21% for GP out-of-hours service—while the number of consultations to GP in-hours service was 13% lower compared to the same time previous year. Increases were observed in calls to NHS 111 for sleep problems.

**Conclusions:**

These analyses showed marked changes in the health care attendances and prescribing for common mental health conditions across a spectrum of health care provision, with some of these changes persisting. The reasons for such changes are likely to be complex and multifactorial. The impact of the pandemic on mental health may not be fully understood for some time, and therefore, these syndromic indicators should continue to be monitored.

## Introduction

Previous infectious disease outbreaks have been shown to worsen mental health [1]. For example, the severe acute respiratory syndrome (SARS) outbreak in 2003 resulted in increased incidence of posttraumatic stress disorder and depressive illness in health care workers [2]. The COVID-19 pandemic has resulted in an unprecedented impact on peoples’ day-to-day lives, with several features potentially adversely affecting mental health. Features include the direct effects of the disease, impact on employment and income, and the prolonged time of restrictions on activities and normal life for the majority of the population.

There is growing research on the size of the impact of COVID-19 on mental health [3-8], much of this from ongoing population surveys using validated mental health scores demonstrating significant impact and that the effect varies across population groups. Young women have been particularly impacted, with higher levels of clinically significant distress [6]. The impacts of previous major coronavirus outbreaks, including the COVID-19 pandemic, on health and social care workers demonstrated a high risk of posttraumatic stress disorder among emergency health care workers [2]. A systematic review of available longitudinal cohort studies concluded a small rise in mental health symptoms immediately after the onset of the pandemic, which dropped to prepandemic levels by mid-2020. A further study examined the impact of the pandemic on primary care–recorded mental health disorders and described a drop in reported illness during March/April 2020. Selected mental health disorders had returned to similar levels by September 2020 in England [3].

However, there is little evidence about how the current pandemic has affected the presentation of mental health conditions to a spectrum of health care settings. We are not aware of work examining the impact of COVID-19 on mental health care usage across multiple health care settings and using routinely available health care data. We hypothesized that common mental health conditions, including depression, anxiety, and sleep disorders, would have been adversely impacted by the first 9 months of the COVID-19 pandemic, and investigated the impact on health care seeking behavior.

Here, we investigated the impact of the COVID-19 pandemic on mental health conditions presenting to a variety of health care services monitored using syndromic surveillance in England. The syndromic surveillance systems use a variety of categorizations to describe the reasons for people presenting, and we tried to identify those presentations that are relevant to mental health. Based on these findings, we proposed a surveillance package of indicators to monitor trends in mental health conditions in real time to provide timely information for action for future events.

## Methods

### Syndromic Surveillance Systems: Background

Syndromic surveillance systems aim to detect outbreaks, to provide situational awareness on the impact of events on the population, and to provide reassurance about the lack of impact of events such as mass gatherings. Real-time syndromic surveillance (using data on patients’ symptoms) is a helpful adjunct to laboratory surveillance and is being used to monitor the impact of COVID-19 on health care–seeking behavior for respiratory illness [9]. The UK Health Security Agency (UKHSA) coordinates a suite of national syndromic surveillance systems that are able to monitor attendances to health care settings in England in near real time (Multimedia Appendix 1, Table S1) [10]. These syndromic surveillance systems are used mainly to monitor the impact of infections (eg, COVID-19 and seasonal influenza) [11,12] and the impact of environmental hazards (eg, heatwaves and flooding). However, the utility of syndromic surveillance systems to monitor changes in the presentation of other diseases or conditions (eg, mental health) in the event of a major incident is being explored.

The primary care database of the Oxford-Royal College of General Practitioners (RCGP) Clinical Informatics Digital Hub (ORCHID) is a database from one of the longest-established primary care sentinel networks worldwide [13,14]. The Oxford RCGP network is able to monitor a wide range of diagnoses, in addition to notifiable diseases and other infections. We used a subset of ORCHID, the Oxford-RCGP Research and Surveillance Centre (RSC) UKHSA COVID-19 Vaccine Effectiveness cohort with good data quality (which was developed to support COVID-19 surveillance [15,16]) to explore recent trends in general practitioner (GP) in-hours consultations for common mental health conditions (Multimedia Appendix 1, Table S1).

### Study Design and Period

We conducted a retrospective observational descriptive study using UKHSA real-time syndromic surveillance systems covering the population of England [12] and the ORCHID GP in-hours data set [16]. We estimated the impact of national lockdown measures using an interrupted time series approach and generalized linear modeling. We extracted data for the period of January 1, 2019, to September 30, 2020.

### Surveillance Data

National Health Service (NHS) 111 calls were extracted from the UKHSA Remote Health Advice syndromic surveillance system. NHS 111 uses “pathways” to triage calls [17]. The data extracted included the number of daily calls that were triaged by the NHS 111 call handlers for the “mental health problems” and “sleep difficulties” pathways and the total number of daily calls in the UKHSA data set. The pathways included in the data set for this study were the first pathway selected by the call handler during the triage process (Multimedia Appendix 1, Table S2).

GP in-hours consultations were based on a total of 504 practices, which included 7,057,447 registered patients during the period of this study. We extracted daily counts of consultations and prescriptions for commonly occurring mental health conditions, including depression and anxiety. Prescriptions included antidepressants, anxiolytics, and hypnotics extracted using lists generated based on the British National Formulary (BNF) [18]. We used a case definition of common mental health problems (CMHPs) developed for the evaluation of community psychology services [19,20], which we subsequently updated from a Read code to the Systematized Nomenclature of Medicine (SNOMED) Clinical Terms [21]. The SNOMED clinical terms are listed in Multimedia Appendix 1, Table S2.

Daily GP out-of-hours consultations were extracted from the UKHSA GP out-of-hours syndromic surveillance system [10,22] for the following: total consultations, all consultations with a clinical (Read) code, consultations with a mental health diagnosis (based on Read code chapter E, “Mental Disorders”; Multimedia Appendix 1, Table S2), consultations for anxiety, and consultations for depression.

The UKHSA National Ambulance Surveillance System (NASS) syndromic data set includes data on specified syndromes and does not represent all ambulance calls. There was no overarching mental health indicator for this system, and thus, we extracted the daily number of ambulance calls for overdoses/ingestion/poisoning based on the chief complaint codes used by the ambulance services (Multimedia Appendix 1, Table S2; we assumed that these were all deliberate overdoses/poisonings but acknowledge that some may have been accidental).

Emergency department (ED) attendances were extracted from the UKHSA Emergency Department Syndromic Surveillance System (EDSSS) for all mental health attendances (as identified in the Emergency Care Data Set diagnosis coding list) [23], acute alcohol intoxication, and drug overdoses (Multimedia Appendix 1, Table S2). In total, 94 type 1 EDs were eligible for inclusion as they had provided data to the UKHSA EDSSS every day for the period of the study.

The diagnosis/triage descriptors were not the same across the syndromic systems. For some of the syndromic surveillance systems (eg, the GP in-hours system), there are validated diagnostic codes describing mental health conditions, whereas for others (eg, NHS 111 and ambulance), calls are based on triage groupings. For each system, we tried to identify an overarching mental health categorization or a description for a condition relevant to mental health (eg, sleep disorders). For each surveillance system included in the study, counts of calls/consultations/attendances were extracted by day and by gender.

### Statistical Analysis

Data were visualized graphically as daily counts and 7-day moving averages (7dma, adjusted for public [bank] holidays) for each of the mental health conditions and surveillance systems from January 1 to September 30, 2019, compared to the equivalent dates in 2020. Data were presented graphically by International Organisation for Standardisation (ISO) week (ISO weeks 1-40).

Data were subdivided into 3 periods: prelockdown (before March 23, 2020), lockdown (March 23-May 31, 2020; ISO weeks 13-22), and postlockdown (June 1-September 30, 2020; ISO weeks 23-40). Generalized linear models (GLMs) were used to model the data, and an interrupted time series approach was used to estimate the impact of national lockdown measures and the changes in health care–seeking behavior since pre- and postlockdown compared to 2019. Count data were modeled using a negative binomial distribution to account for overdispersion, which is common in health data. Systematic differences in the daily data caused by weekends and public holidays were accounted for by including a binary variable for working days versus weekends and public holidays. Annual seasonality was modeled by including a harmonic term using Fourier transforms. For each of the 3 periods (pre-, during, and postlockdown), variables were included to model step changes and trends separately. The resulting models were compared with the actual data, and the residuals for signs of bias were checked.

To estimate the impact of lockdown and changes postlockdown, GLMs were used to create counterfactual models of what would have been expected if the pandemic and lockdown had not occurred. The lockdown period (March 23-May 31, 2020) was characterized by a sudden sharp decrease in health care–seeking activity, followed by an increasing trend; therefore, the estimate for the impact of lockdown was based on a single date (March 23, 2020) to show the full extent of the impact. Postlockdown (June 1-September 30, 2020), trends were more stable, so comparing average activity across the whole period provided an estimate for the longer-term impacts. First, the actual data on March 23, 2020, were compared with the counterfactual model for March 23, 2020, setting the variables for the step change and trend during lockdown, to lockdown not having occurred. Second, to estimate how activity has changed postlockdown compared to what we would expect at this time of year, actual activity postlockdown was compared with the counterfactual model.

The advantage of using an interrupted time series approach over simply comparing with the previous year’s data is that we could account for any long-term trends and lessen the impact of any short fluctuations in data that would make 2019 incomparable with 2020, thus providing less biased estimates for the direct effects of lockdown. To provide 95% CIs around our estimates for the change in postlockdown activity, a bootstrap method was used to calculate the bias-corrected and accelerated bootstrap interval. The model and formulae used are included in Multimedia Appendix 1.

All statistical analyses were completed in R software (R Foundation for Statistical Computing) using the *Modern Applied Statistics with S* (*MASS*), *tsModel*, and *boot* packages [24-28].

### Ethical Considerations

All data used in this study were anonymized. The UKHSA has access to a range of data sources under Regulation 3 (Health Protection) of the Health Service (Control of Patient Information) Regulations 2002. The use of ORCHID data was specifically approved by the UKHSA Caldicott Guardian as an addendum to the data sharing agreement with the University of Oxford. Patients or the public were not involved in the design, conduct, reporting, or dissemination plans of our research.

## Results

### Calls/Consultations/Attendances

From January 1, 2019, to September 30, 2020, the syndromic data included 25,718,106 total calls to NHS 111 (an average of 40,247 daily calls); 1,427,507 GP in-hours mental health consultations (including telephone consultations) in the sentinel network (an average of 2199 daily consultations); 16,090,272 total GP out-of-hours consultations (an average of 25,180 daily consultations), of which 6,307,387 (39.2%) had a clinical code; 9,284,990 total ambulance calls (an average of 14,531 daily calls); and 13,821,306 total ED attendances (an average of 21,630 daily attendances). These figures represent the data routinely available through the syndromic surveillance systems, though coverage of England for each of the systems varies (Multimedia Appendix 1, Table S3).

### All Mental Health Presentations and GP Prescriptions for Mental Health Medications

**Figure 1:**
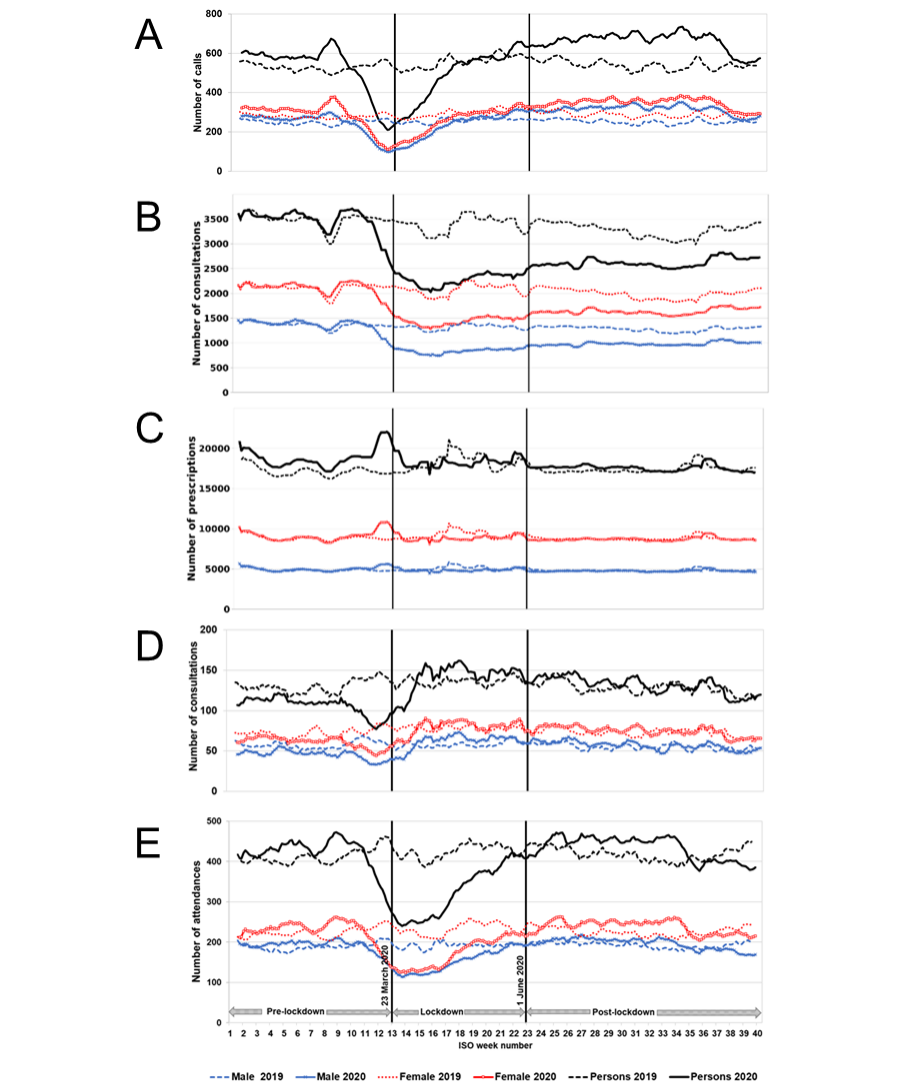
Calls, consultations, and attendances for mental health conditions presenting to NHS 111, GP in-hours and GP out-of-hours and EDs, and GP in-hours mental health medications in comparison to selected key dates in the pandemic. (A) NHS 111 calls for mental health problems, (B) GP in-hours consultations for mental health conditions, (C) GP in-hours prescriptions for mental health medications, (D) GP out-of-hours consultations for all mental health conditions, and (E) ED attendances for mental health conditions. Daily calls/consultations/attendances/prescriptions presented as 7dma adjusted for bank holidays (BH) and by gender. The start of lockdown (March 23, 2020) and the start of the postlockdown period (June 1, 2020) are indicated by vertical lines. 7dma: 7-day moving averages; ED: emergency department; GP: general practitioner; ISO: International Organisation for Standardisation; NHS: National Health Service.

**Figure 2:**
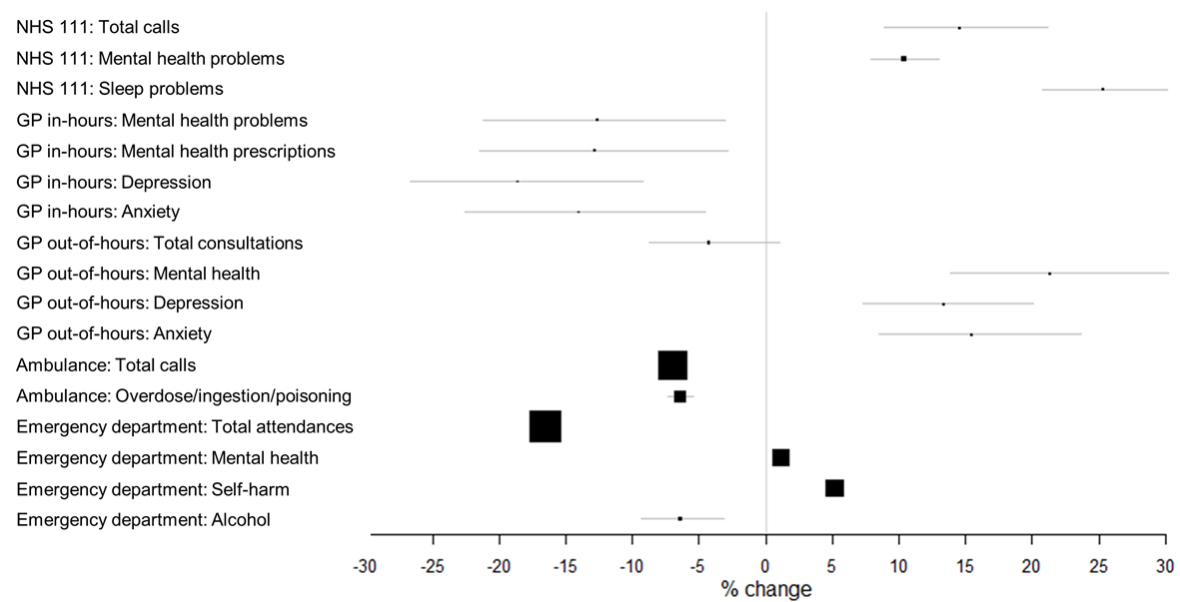
Summary of changes in syndromic indicators for the postlockdown period across systems compared to that expected. GP: general practitioner; NHS: National Health Service.

Calls to NHS 111 triaged using the mental health problem pathways occurred at a slightly increased level at the beginning of 2020 compared to the same time in 2019 and showed an initial peak in mid-February 2020 (ISO week 8); see Figure 1A. Call numbers thereafter decreased to the lowest level on March 19, 2020 (ISO week 12), just before the lockdown commenced (on March 23, 2020; Table 1), and then increased throughout the lockdown and remained elevated throughout the postlockdown period (Figure 1A). Call levels, as estimated by the interrupted time series model, during the postlockdown period (June 1-September 30, 2020) were approximately 10% above expected levels of the counterfactual model (additional daily mean of 62 calls; 95% CI 51-73; Figure 2 and Table 2).

GP in-hours consultations for all mental health conditions began to drop sharply in the week commencing March 2, 2020 (ISO week 10), and continued to fall until the week commencing April 6, 2020 (ISO week 15), when consultations started to rise again, though they remained at reduced levels (Figure 1B). Mean daily levels of GP in-hours consultations for all mental health conditions reduced by 13% in the postlockdown period (June 1-September 30, 2020) compared to those modeled if the pandemic had not occurred (Table 2). Consultations during the whole period were higher in females compared to males (Figure 1B). GP in-hours prescriptions for mental health medications showed a sharp spike just prior to lockdown (Figure 1C), increasing by 27% on March 23, 2020, compared to those expected if the pandemic had not occurred (Table 1), and reduced by 13% compared to those expected for the postlockdown period (Table 2).

Mean daily GP out-of-hours consultations for all mental health conditions occurred at a slightly reduced level at the beginning of 2020 compared to 2019 and then started to decrease from late February 2020 (ISO week 9) to levels on March 23, 2020, approximately 12% below that expected from the model (Table 1). Levels subsequently started to increase, and postlockdown remained elevated until early June (ISO week 23), after which the levels were similar to 2019 (Figure 1D). Mean daily levels of GP out-of-hours consultations for all mental health conditions increased by 21% compared to those expected in the postlockdown period (June 1-September 30, 2020), with an additional daily mean of 23 consultations (95% CI 19-27; Table 2).

ED attendances for all mental health diagnoses occurred at slightly higher levels during the first part of 2020 compared to 2019 (Figure 1E) and, as for other systems, decreased during March and remained low for the first half of the lockdown period (Figure 1E). The number of attendances on March 23, 2020, was 38% below that expected from the counterfactual model (Table 1). Following the period of lockdown (March 23-May 31, 2020), levels returned to those similar to those expected (Figure 2 and Table 2), while total ED attendances reduced by 17%.

**Table 1:** Interrupted time series analysis illustrating a comparison of modeled versus measured call, consultation, attendance, and prescription counts presenting to a number of health care systems: NHS^a^ 111, GP^b^ in-hours and out-of-hours consultations, ambulance services, and EDs^c^ on the first day of lockdown (March 23, 2020).

System and syndrome		Modeled (ie, if pandemic had not happened) number on first day of lockdown	Actual number on first day of lockdown	Estimated change on first day of lockdown (actual number – modeled number)	Percentage change (%)
**NHS 111 calls**					
	Total calls	33,104	37,572	4468	13
	Mental health problems	550	214	336	–61
	Sleep difficulties	24	12	12	–49
**GP in-hours consultations or prescriptions**					
	Mental health problems	3155	2859	296	–9
	Mental health prescriptions	20,639	26,137	5496	27
	Depression	1560	1421	139	–9
	Anxiety	1428	1420	–8	–1
**GP out-of-hours consultations**					
	Total consultations	20,861	20,628	–233	–1
	Mental health	87	76	–11	–12
	Depression	18	7	–11	–60
	Anxiety	49	51	2	4
**Ambulance calls**					
	Total syndromic calls	14,705	17,156	2451	17
	Overdose/ingestion/poisoning	509	362	–147	–29
**ED attendances**					
	Total attendances	23,758	13,191	–10,567	–44
	Mental health	434	269	–165	–38
	Overdose	182	97	–85	–47
	Excess alcohol use	162	86	–76	-47

^a^NHS: National Health Service.

^b^GP: general practitioner.

^c^ED: emergency department.

**Table 2:** Interrupted time series analysis illustrating a comparison of modeled versus measured call, consultation, attendance, and prescription counts presenting to a number of health care systems: NHS^a^ 111, GP^b^ in-hours and out-of-hours consultations, ambulance services, and EDs^c^ during the postlockdown period (June 1-September 30, 2020).

System and syndrome		Modeled (ie, if pandemic had not happened) daily mean number in postlockdown period, estimate (95% CI)	Actual daily mean number in postlockdown period	Estimated difference due to pandemic in daily mean postlockdown period, estimate (95% CI)	Percentage change (%)
**NHS 111 calls**					
	Total calls	37,606 (35,553-39,532)	43,071	5465 (4653-6295)	15
	Mental health problems	599 (585-613)	661	62 (51-73)	10
	Sleep difficulties	27 (26-28)	34	7 (5-8)	25
**GP in-hours consultations or prescriptions**					
	Mental health problems	2178 (1963-2414)	1903	–275 (–317 to –232)	–13
	Mental health prescriptions	14,592 (13,093-16,197)	12722	–1870 (–2342 to –1392)	–13
	Depression	1090 (978-1210)	887	–202 (–229 to –176)	–19
	Anxiety	1022 (920-1134)	878	–144 (–166 to –122)	–14
**GP out-of-hours consultations**					
	Total consultations	24,444 (23,149-25,634)	23,391	–1053 (–1562 to –512)	–4
	Mental health	109 (101-116)	132	23 (19-27)	21
	Depression	20 (18-21)	22	3 (2-4)	13
	Anxiety	62 (58-66)	71	10 (7-12)	15
**Ambulance calls**					
	Total syndromic calls	14,883 (14,827-14,938)	13,842	–1041 (–1194 to –889)	–7
	Overdose/ ingestion/ poisoning	571 (566-577)	535	–37 (–45 to –28)	–6
**ED attendances**					
	Total attendances	23,865 (23,776-23,959)	19,925	–3940 (–4201 to –3681)	–17
	Mental health	428 (426-431)	433	5 (–1 to 11)	1
	Overdose	179 (178-180)	188	9 (6-12)	5
	Excess alcohol use	198 (192-205)	186	–13 (–17 to –8)	–6

^a^NHS: National Health Service.

^b^GP: general practitioner.

^c^ED: emergency department.

### Depression

**Figure 3:**
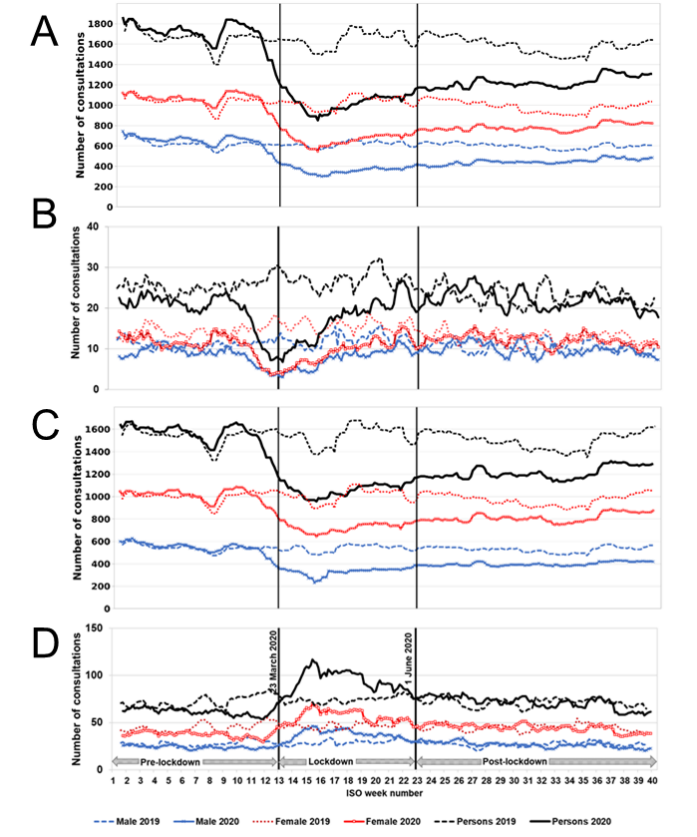
Consultations for depression and anxiety presenting to GP in-hours and out-of-hours in comparison to selected key dates in the pandemic. (A) GP in-hours consultations for depression, (B) GP out-of-hours consultations for depression, (C) GP in-hours consultations for anxiety, and (D) GP out-of-hours consultations for anxiety. Daily consultations presented as 7dma adjusted for bank holidays (BH) and by gender. The start of the lockdown (March 23, 2020) and the start of the postlockdown period (June 1, 2020) are indicated by vertical lines. 7dma: 7-day moving averages; GP: general practitioner; ISO: International Organisation for Standardisation.

GP in-hours consultations for depression showed a similar pattern to all mental health conditions (Figure 3A). Mean daily levels of GP in-hours consultations for depression showed a decrease of 19% in the postlockdown period (June 1-September 30, 2020) compared to that expected had the pandemic not occurred (Table 2). GP out-of-hours consultations for depression showed a similar pattern to all mental health conditions (Figure 3B). Mean daily levels of GP out-of-hours consultations for depression showed an increase of 13% in the postlockdown period, although daily numbers were small (Figure 2 and Table 2).

### Anxiety

GP in-hours consultations for anxiety reduced as the lockdown approached with the introduction of social distancing measures and remained below levels seen in 2019 for the remainder of the study period (Figure 3C). In the postlockdown period (June 1-September 30, 2020), total consultations for anxiety were 14% below modeled expected levels if the pandemic had not occurred (Figure 2 and Table 2). GP out-of-hours consultations for anxiety were below levels seen in 2019 but relatively stable until mid-March (ISO week 11), after which levels rose until a peak on April 9, 2020 (ISO week 15; Figure 3D). Overall anxiety consultations remained 15% above expected levels (had the pandemic not occurred) during the postlockdown period (Figure 2 and Table 2). GP consultations (in-hours/out-of-hours) for anxiety were higher in females than in males in both 2019 and 2020 (Figures 3C and 3D).

### Sleep Difficulties

**Figure 4:**
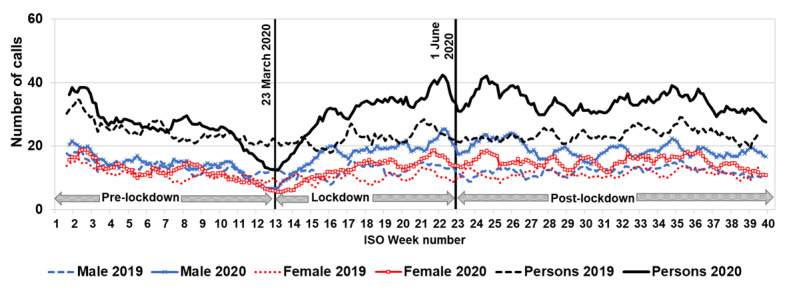
Calls to NHS 111 for sleep difficulties in comparison to selected key dates in the pandemic. Daily numbers of calls presented as bank holiday (BH)-adjusted 7dma and by gender. The start of the lockdown (March 23, 2020) and the start of the postlockdown period (June 1, 2020) are indicated by vertical lines. 7dma: 7-day moving averages; ISO: International Organisation for Standardisation; NHS: National Health Service.

Calls to NHS 111 triaged for sleep difficulties fell sharply in January 2020, a trend also seen in January 2019. Calls to NHS 111 for sleep difficulties rose slightly in mid-February 2020 (ISO week 8; Figure 4) and then reduced to a low of approximately 50% of expected levels at the start of lockdown on March 23, 2020 (Table 1). Thereafter, calls for sleep difficulties increased to 25% above modeled expected levels in the postlockdown period (June 1-September 30, 2020; Table 2). Calls for sleep difficulties for males were higher than those for females, but calls by both genders peaked just before lockdown easing commenced at the beginning of June 2020 (ISO week 23; Figure 4).

### Measures of Self-Harm

#### Overdose

**Figure 5:**
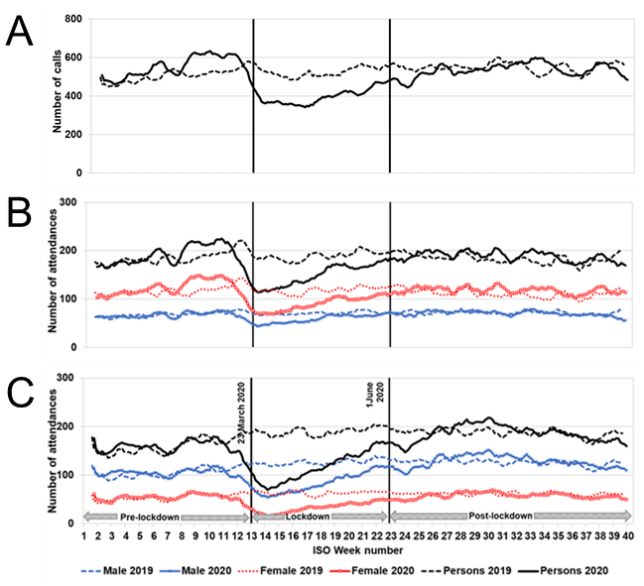
Ambulance calls and ED attendances for indicators of self-harm (overdose and excess alcohol use) in comparison to selected key dates in the pandemic. (A) Ambulance calls for overdose, (B) ED attendances for overdose, and (C) ED attendances for excess alcohol use. Daily numbers of call-outs/attendances presented as bank holiday (BH)-adjusted 7dma and by gender (ED only). The start of lockdown (March 23, 2020) and the start of the postlockdown period (June 1, 2020) are indicated by vertical lines. 7dma: 7-day moving averages; ED: emergency department; ISO: International Organisation for Standardisation.

From January 2020 to the announcement of stay-at-home and social distancing advice on March 11, 2020 (ISO week 11), ambulance calls for overdose/ingestion/poisoning increased and then sharply decreased until the start of lockdown (March 23, 2020; Figure 5A), when calls reduced by 29% compared to those expected from the model (Table 1). From the start of lockdown, the number of calls gradually increased, and during the postlockdown period (June 1-September 30, 2020), calls were slightly reduced at 6% lower than estimated had the pandemic not occurred (37 fewer mean daily call-outs; 95% CI –45 to –28; Table 1).

Attendances at EDs for overdoses markedly increased during January and February 2020 (particularly in females) compared to 2019 (Figure 5B). Attendances showed a sharp drop following the introduction of social distancing advice on March 11, 2020 (ISO week 11), and by the start of lockdown (March 23, 2020), levels reduced by 47% compared to those expected using the model (Table 1). This was followed by a gradual increase to levels similar to 2019 (and for both genders) during the postlockdown period (June 1-September 30, 2020; Figure 5B).

#### Excess Alcohol Use

Attendances to EDs for excess alcohol use showed a drop following the introduction of social distancing advice in early March 2020 (ISO week 11) and continued to drop at the start of lockdown (March 23, 2020; Figure 5C). The interrupted time series model estimated that there were 47% fewer attendances than expected on March 23, 2020 (Table 1). During lockdown (March 23-May 31, 2020), there was a gradual increase, with levels postlockdown (June 1-September 30, 2020) only slightly reduced (6%) compared to those expected using the model (Table 2). Attendances were consistently higher in males than in females.

## Discussion

### Principal Findings

Looking across the health care systems, all showed an initial dip in attendance for mental health conditions after the introduction of social distancing advice in early March 2020 and the first lockdown and then increased. This pattern mirrored total (all cause) activity in each system and attendances for other non-COVID-19 conditions [10]. For NHS 111 and GP out-of-hours services, mental health activity levels postlockdown increased (by 10% for NHS 111 and 21% for GP out-of-hours services). The levels of GP in-hours consultations for mental health remained approximately 13% lower compared to modeled levels expected if the pandemic had not occurred. It is possible that there has been a shift in consulting on mental health conditions from GP in-hours services to other health services, such as NHS 111 and GP out-of-hours services.

GP in-hours health care contacts for depression mirrored those for all mental health attendances, showing a decrease during the prelockdown (before March 23, 2020) and lockdown periods and then returning to levels approximately a fifth lower than those expected. GP out-of-hours health care contacts for depression mirrored those for other attendances, showing a decrease during the prelockdown (before March 23, 2020) and lockdown periods and then returning to levels about 13% increased to those expected (although daily numbers were low).

The number of GP contacts for anxiety showed different patterns in-hours and out-of-hours. GP in-hours contacts decreased and remained 14% lower compared to those expected during the postlockdown period. GP out-of-hours health care contacts for anxiety increased during lockdown and remained at about 15% above expected levels during the postlockdown period.

Health care contacts to NHS 111 for sleep disorders increased during lockdown and then remained elevated until the end of the study period. Daily numbers of calls to NHS 111 about sleep difficulties increased by approximately a quarter in the postlockdown period to those expected; thus, there was a persisting and notable continuing impact.

### Surveillance of Mental Health During COVID-19

The COVID-19 pandemic has resulted in several surveillance initiatives to monitor the impact of the pandemic on mental health. The UKHSA publishes a regular overview of such impact (particularly using population surveys, longitudinal studies, and results from academic studies) [29]. Analysis using the Clinical Practice Research Datalink (CPRD) showed similar trends as our study, with a marked reduction in GP in-hours consultations for a variety of mental health conditions and a persisting impact with reduced levels of consultations lasting until July 2020 [5]. The authors used an interrupted time series approach using weekly data, taking the exposure as the introduction of lockdown and comparing back to 2017. The authors described the likely unmet need for mental health services and highlighted the need to prepare for increased demand. Reports from the Nuffield Trust and the NHS Confederation found evidence that fewer people were able to access mental health services during the first lockdown. New referrals for treatment and support for common mental health conditions, such as depression and anxiety, provided by the Improving Access to Psychological Therapies (IAPT) program fell by 61% over the first lockdown [30,31]. This was reflected in a survey of 130 countries by the World Health Organization (WHO) during June-August 2020, which reported widespread disruptions to many critical mental health services [32,33].

A further study used primary care electronic health records to examine the impact of the pandemic on mental health conditions presenting to GPs and showed a drop in reported illness during March/April 2020. Selected mental health disorders had returned to similar levels by September 2020 in England; however, the rates of incident depression and anxiety disorder remained a third lower in the rest of the United Kingdom (UK), consistent with the sustained reduction we noted in presentations to GP in-hours consultations [3].

Results from 2 longitudinal UK population cohorts showed that anxiety and lower well-being, but not depression, increased during the COVID-19 pandemic compared to prepandemic assessments. The percentage of individuals with probable anxiety disorder was almost double during the COVID-19 pandemic [4]. Our study, focusing on health care–seeking behavior, showed similar increases in anxiety presenting to GP out-of-hours service but, conversely, reductions in anxiety presenting to GP in-hours service (again likely reflecting the overall reduction in people presenting for all causes).

The marked and continuing impact of the pandemic on good sleep is described in other studies (our work suggesting a 25% increase in calls, as monitored by NHS 111); in the UK, those experiencing sleep problems increased from 16% to 25% in April 2020 [29]. In Italy, during the period of lockdown, 42% people reported sleep disturbances, with 17% [34] disturbances described as moderate or severe; and in a cross-sectional survey in France, 19% people were categorized as having insomnia [35]. A study in the United States using ED syndromic surveillance showed a similar reduction in consulting for a variety of mental health conditions in early March, but the median visit rates for suicide attempts and overdoses for the period of mid-March 2020 to October 2020 were higher than the rates for the same period in 2019 [36]. Finally, real-time surveillance used Google trend data to assess the impact of the pandemic on mental health in the United States, identifying pandemic-associated spikes in anxiety [37].

### Syndromic Surveillance of Mental Health Following Incidents

There are examples of syndromic surveillance systems being used to monitor the impact on mental health after public health incidents. Such systems have been predominantly using a single data source rather than across health care services and include the use of ED [38] and Twitter (social media) [39] analysis following terrorist attacks in France. ED surveillance for mental health in New York State was conducted post–Hurricane Sandy [40], and ED surveillance of attendances for mental health and substance use presenting to Californian EDs concluded that mental health data from syndromic systems are uniquely available in real time as an indicator of service utilization and thus particularly useful for emergency events [41].

Although not included in our study, the Improved Access to Psychological Therapies (IAPT) program in England also offers a service to people with CMHPs. IAPT principally offers cognitive behavioral therapies, and people can be referred or can self-refer. IAPT reports a reduction in referral (including self-referral), entering and completing therapy, postlockdown (Multimedia Appendix 1, Figure S1) [42].

### Strengths and Limitations

This work has several strengths. It describes impact on health care–seeking behavior for mental health conditions across a variety of health care provisions ranging from NHS telephone help lines to ED attendances. The surveillance systems used here are well established and cover England (although several are sentinel systems). Such diversity of surveillance systems enables us to triangulate and describe both consistent trends across systems and to look for changes in severity.

The multiple health care systems on which these surveillance systems are based use various coding systems/triage mechanisms, and thus, we have established different data sets but similar diagnostic/syndromic groupings to enable a multiple cross-condition “snapshot” for monitoring the impact of future major public health incidents. Although we analyzed these data retrospectively, we now have a “common mental health” presentation surveillance package, including an ontology of relevant codes across the multiple systems, which can be prospectively incorporated into routine monitoring, thus enabling the real-time use for mental health surveillance with validated baselines for future events. Such a suite of indicators will provide timely information for incident directors and those managing incidents as to where resources may be needed at the time of an incident and subsequently. Such analyses could additionally include assessing the impact by age, sex, severity, and geography. These data are available in near real time (daily except for the ORCHID system, which is twice weekly), and further work includes establishing which of the indicators are most useful in assessing the impact of differing types of incidents (eg, epidemics, deliberate incidents, and flooding) and exploring inequalities in access.

There are, however, several limitations to this work: The changes in health care provision and guidance issued to the public on which services to use during the pandemic will have impacted on established baselines, causing difficulties in interpretation of changes in consulting. For example, the observed change in consulting numbers may have been driven either by true changes in incidence or by the national advice not to consult in person with a GP. It is also possible that other changes in the scheduled GP service, such as greater use of text messaging or online consultations, may have meant that not all encounters were captured or be recorded as “clinical administration” within the GP computerized medical record. The move to 15-minute appointments may have also contributed to the fall in consultation numbers [43].

Using routinely available health care data, it is difficult to disentangle true changes in the incidence of mental health conditions from the effect of public health messaging, health care–seeking behavior, and changes in health care provision. These multiple and complex drivers of change have made interpretation of surveillance data difficult during the COVID-19 pandemic [11]. We focus here on the cross–health care usage for syndromes associated with mental health and describe trends, rather than directly inferring changes in community incidence.

The changing trends we have observed are likely to reflect the “tip of the iceberg” in terms of mental health impact on the community. It is known that most patients with mental health conditions or poor well-being are likely to self-care or not seek help from a health care provider [44-46]. Further work is needed to understand the impact of this pandemic on mental health and well-being. This work has established a surveillance package that can be applied to routine public health surveillance programs to undertake real-time surveillance of mental health presentations during future major health protection incidents.

### Conclusion

These analyses showed marked changes in the health care attendances and prescribing for common mental health conditions, across a spectrum of health care provision, with some of these changes persisting. The reasons for such changes are likely to be complex and multifactorial. The impact of the pandemic on mental health may not be fully understood for some time, and therefore, these syndromic indicators should continue to be monitored.

## Appendix

Multimedia Appendix 1 Supplementary file including tables, figures, and scripts.

## Abbreviations

7dma: 7-day moving averages
CMHP: common mental health problem
ED: emergency department
EDSSS: Emergency Department Syndromic Surveillance System
GLM: generalized linear model
GP: general practitioner
IAPT: Improved Access to Psychological Therapies
ISO: International Organisation for Standardisation
NHS: National Health Service
ORCHID: Oxford-Royal College of General Practitioners Clinical Informatics Digital Hub
RCGP: Royal College of General Practitioners
SNOMED: Systematized Nomenclature of Medicine
UKHSA: UK Health Security Agency

## Data Availability

The data sets used in this study are not publicly available. The aggregated data used for the time series analyses can be made available on request through the UK Health Security Agency (UKHSA) Office for Data Release. The Oxford-Royal College of General Practitioners Clinical Informatics Digital Hub (ORCHID) data can be accessed by researchers. Approval is on a project-by-project basis. Ethical approval by a UK National Health Service Research Ethics Committee is needed before any data release or other appropriate approval. Researchers wishing to directly analyze patient-level pseudonymized data will be required to complete information governance training and work on the data from the secure servers at the University of Oxford. Patient-level data cannot be taken out of the secure network. We encourage interested researchers to attend the short courses on how to analyze primary care or Royal College of General Practitioners (RCGP) Research and Surveillance Centre data, which are open to enrolment twice a year.
