# Supplementary material for: Mental Health Presentations Across Health Care Settings During the First 9 Months of the COVID-19 Pandemic in England: Retrospective Observational Study
Source: JMIR Public Health Surveill. 2022 Aug 3;8(8):e32347. doi: 10.2196/32347 (PMC9359118; doi:10.2196/32347)
Supplement: Multimedia Appendix 1 [file publichealth_v8i8e32347_app1.docx]

**Multimedia Appendix 1**

**Table S1:** Description of the syndromic surveillance systems used in the study

| **System** | **Coverage** | **Description** | **Caveats** |
| --- | --- | --- | --- |
| **UKHSA Remote Health Advice Syndromic Surveillance System (NHS 111 calls)** | National (England) | A UKHSA syndromic surveillance system which uses fully anonymised, daily data on calls to NHS 111 (England) | Calls to NHS 111 triaged using NHS Pathways. The syndromic dataset excludes calls to NHS 111 where an immediate threat to life was identified and other calls that have not been triaged using NHS Pathways (this may include calls for repeat prescriptions, information etc). The data are sourced from the NHS 111 Repeat Caller Database |
| **ORCHID primary care dataset of the Royal College of General Practitioners (RCGP) Research and Surveillance Centre (RSC) sentinel network** | Sentinel (England) | A network of general practices, which extracts data from the computerised medical record systems of over 500 practices in England. This network provides a representative sample of the population of England in terms of demographics and clinical outcomes^a^ | Data included in this study includes all consultations, diagnoses and symptoms for all common mental health conditions recorded in electronic healthcare records (EHRs) in the network of practices. <https://orchid.phc.ox.ac.uk/index.php/orchid-data/>  GP in-hours consultations were based upon a total of 504 practices, which included 7,057,447 registered patients during the period of this study |
| **UKHSA GP Out-of-Hours Syndromic Surveillance System** | Sub-national (England) | Pseudo-anonymised data uploaded from GP out-of-hours service providers who use the Advanced Health and Care Adastra software system and who have agreed to participate in the UKHSA GP out-of-hours system | Only consultation data for 28 out-of-hours service providers that reported consistently throughout the study period were included in the study. Clinical coding varies across the out-of-hours service providers and in this dataset only 39% of consultations had a clinical code |
| **UKHSA National Ambulance Syndromic Surveillance System** | National (England) | All ambulance trusts in England reporting daily data to the UKHSA NASSS | Total syndromic calls included all calls where the chief presenting complaint was mapped to one of the syndromic indicators monitored by UKHSA. This does not include all calls submitted to the ambulance trusts. 10 ambulance trusts were included in this study |
| **UKHSA Emergency Department Syndromic Surveillance System** | **National (England)** | UKHSA national EDSSS began operating in April 2018 following the introduction of the  Emergency Care Data Set (ECDS).^b^ Not all EDs currently provide data through ECDS on a daily basis | 94 Type 1 ED’s which reported consistently for the whole of the study period were included in this study. The indicators analysed in this study are based on the primary diagnosis  of each attendance as reported by EDs using SNOMED CT codes |

^a^<https://orchid.phc.ox.ac.uk/> ; <https://bmjopen.bmj.com/content/6/4/e011092>

^b^<https://digital.nhs.uk/data-and-information/data-collections-and-data-sets/data-sets/emergency-care-data-set-ecds>

**Table S2:** Description of the mental health indicators used in the study

| **System** | **Group/condition** | **Pathway/**  **Read code/**  **SNOMED code** | **Description of indicator** |
| --- | --- | --- | --- |
| **NHS 111 calls** | Mental Health Problems | Pathway | Pathway describing known mental health problems |
| **NHS 111 calls**  **GP in-hours consultations (ORCHID)** | Sleep difficulties | Pathway | Sleep difficulties |
|  | Total mental health | SNOMED CT | Mental Disorder |
| **GP in-hours consultations (ORCHID)**  **GP out-of-hours consultations** | Prescribing | British National Formulary (BNF78, 2019-2020, bnf.org) | Pharmacological groups such as antidepressants, anxiolytics and hypnotics |
|  | Depression | SNOMED CT | Depressive disorder; organic mood disorder; mild major depression single episode; mild recurrent major depression; moderately severe major depression single episode; moderate major depression single episode; moderate recurrent major depression; postpartum depression; atypical depression; post-schizophrenic depression; psychosis and severe depression with and due to bpd; schizoaffective disorder depressive type; severe major depression with anxiety single episode; severe recurrent major depression; severe recurrent major depression with psychotic feature; single major depressive episode severe with psychosis; bipolar affective disorder current episode depression |
|  | Anxiety | SNOMED CT | Anxiety disorder; organic anxiety disorder; generalised anxiety disorder; illness anxiety disorder; panic disorder; post-traumatic stress disorder; obsessive-compulsive disorder |
|  | All Mental Health Disorders | Read code | Based on Read code Chapter E Mental disorders |
| **GP out-of-hours consultations**  **Ambulance calls** | Depression | Read code | Depressed; symptoms of depression; low mood; depressive episodes; post viral depression; dysthymia; restlessness and agitation |
|  | Anxiety | Read code | Anxiousness; life crisis; generalised anxiety disorder; acute reaction to stress; panic attacks; Chronic post-traumatic stress disorder |
|  | Overdose/ Ingestion/ Poisoning |  | Overdoses, ingestion of a substance or poisoning |
| **Emergency department attendances** | Mental health | SNOMED | Dementia; delirium; personality disorder; eating disorder; depressive disorders; anxiety; psychotic disorders; bipolar disorders; schizophrenia; somatization disorder; somatoform disorder; dissociative disorder; adjustment disorder; factitious disorder |
| **Emergency department attendances** | Overdose | SNOMED | Acetaminophen overdose; Non-steroidal anti-inflammatory overdose; overdose of antidepressant drug; sedative overdose; overdose of opiate |
|  | Alcohol | SNOMED | Alcohol intoxication; alcohol dependence; Uncomplicated alcohol withdrawal; Toxic effect of alcohol; Alcohol withdrawal induced convulsion |

**Table S3:** Total calls/consultations and attendances by system during the study period (January 1, 2019 to September 30, 2020)

| **System** | **Total calls/consultations/attendances between 1 January 2019 and 30 September 2020 (average daily)** |
| --- | --- |
| NHS 111 calls | 25,718,106 (40,247) |
| GP in-hours consultations^a^ | 1,427,507 (2,199) |
| GP out-of-hours consultations | 16,090,272 (25,180) |
| Ambulance calls^b^ | 9,284,990 (14,531) |
| Emergency department attendances | 13,821,306 (21,630) |

^a^ mental health consultations only

^b^ total syndromic ambulance calls only

**Supplementary figure 1:** Improved Access to Psychological Therapies (IAPT) programme activity, England, 2020.


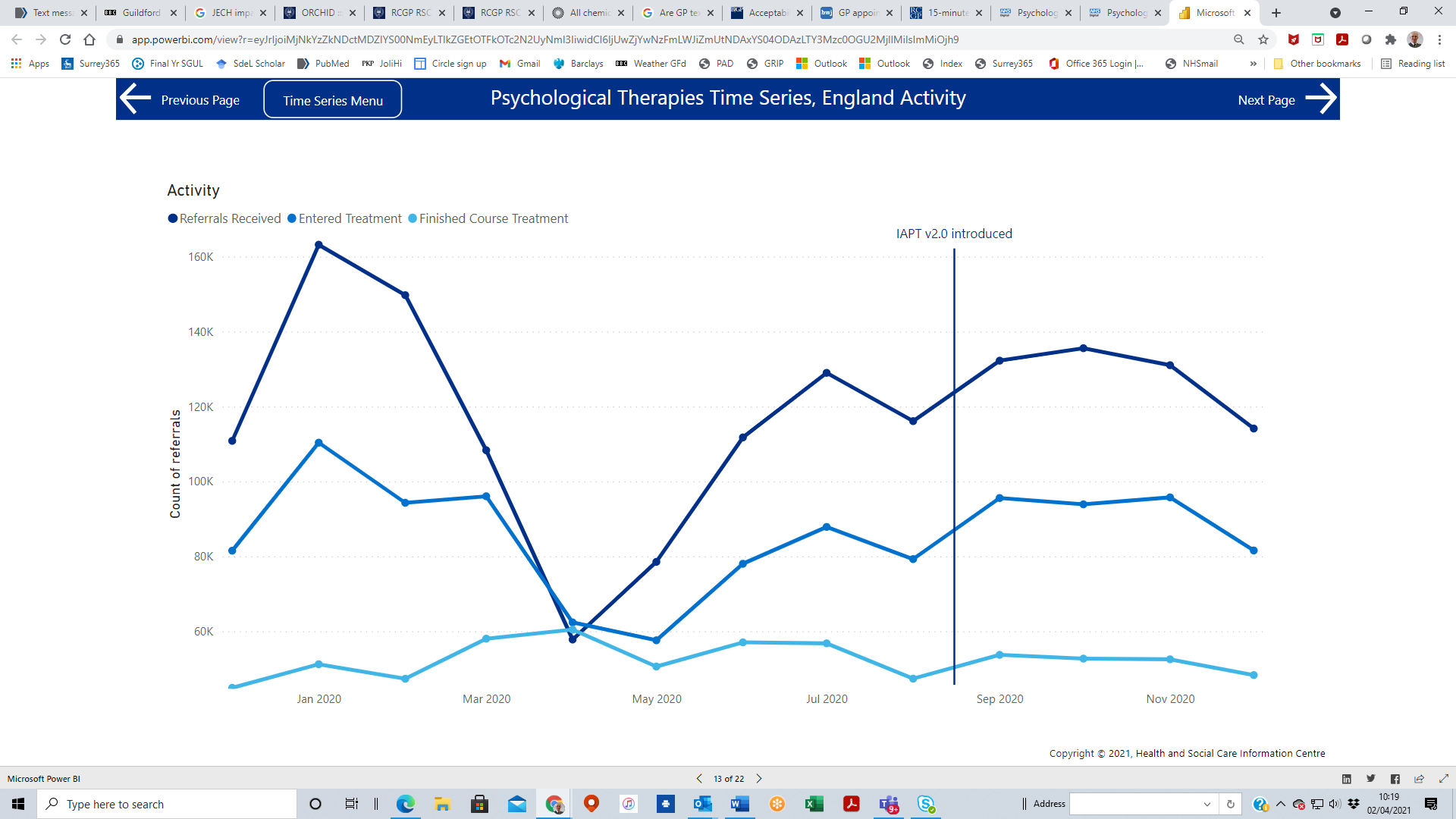


Source: <https://digital.nhs.uk/data-and-information/publications/statistical/psychological-therapies-report-on-the-use-of-iapt-services>

**Supplementary model script**

**Example R code used for interrupted time series models**

model <- glm.nb(MHcalls ~ holiday + harmonic(week,1,52) + trend

+ pre-lockdown + pre-lockdown* trend + pre-lockdown* trend*trend

+ lockdown + lockdown* trend + lockdown* trend*trend

+ post-lockdown+ post-lockdown* trend + post-lockdown* trend*trend

, data=model_data)

Variable key:

MHcalls: the dependent variable – a count of the mental health indicator

Holiday: A variable that was 1 for weekends and public holidays, otherwise 0

Week: the numerical week number

Trend: date expressed as a number

Pre-lockdown, lockdown and post-lockdown: binary variables used to define whether a date was in the “pre-lockdown” (January 1, 2019 - March 22, 2020), “lockdown” (March, 23 – May 31, 2020) or “post-lockdown” (June 1 – September 30, 2020) period.

Modelling was undertaken in R using the MASS and tsModel packages

To cite R in publications, use:

R Core Team (2021). R: A language and environment for statistical computing. R Foundation for Statistical Computing, Vienna, Austria. URL https://www.R-project.org/.

A BibTeX entry for LaTeX users is:

@Manual{,

title = {R: A Language and Environment for Statistical Computing},

author = {{R Core Team}},

organization = {R Foundation for Statistical Computing},

address = {Vienna, Austria},

year = {2021},

url = {https://www.R-project.org/},

}

To cite the MASS package in publications, use:

Venables, W. N. & Ripley, B. D. (2002) Modern Applied Statistics with S. Fourth Edition. Springer, New York. ISBN 0-387-95457-0

A BibTeX entry for LaTeX users is:

@Book{,

title = {Modern Applied Statistics with S},

author = {W. N. Venables and B. D. Ripley},

publisher = {Springer},

edition = {Fourth},

address = {New York},

year = {2002},

note = {ISBN 0-387-95457-0},

url = {https://www.stats.ox.ac.uk/pub/MASS4/},

}

To cite package ‘tsModel’ in publications use:

Roger D. Peng and with contributions from Aidan McDermott (2013). tsModel: Time Series Modeling for Air Pollution and Health. R package version 0.6. https://CRAN.R-project.org/package=tsModel

A BibTeX entry for LaTeX users is:

@Manual{,

title = {tsModel: Time Series Modeling for Air Pollution and Health},

author = {Roger D. Peng and with contributions from Aidan McDermott},

year = {2013},

note = {R package version 0.6},

url = {https://CRAN.R-project.org/package=tsModel},

}
